# Localized Mechanical Actuation using *pn* Junctions

**DOI:** 10.1038/s41598-019-49988-z

**Published:** 2019-10-16

**Authors:** Mikhail Kanygin, Abbin Perunnilathil Joy, Behraad Bahreyni

**Affiliations:** School of Mechatronic Systems Engineering, Simon Fraser University, Surrey, BC V3T 0A3 Canada

**Keywords:** NEMS, Electrical and electronic engineering, Electronics, photonics and device physics

## Abstract

We are reporting on the fabrication and characterization of microscale electromechanical actuators driven by the internal forces induced within the depletion region of a typical *pn* junction. Depletion region actuators operate based on the modulation of the interactions of the internal electric field and the net space charge within the depletion region of a *pn* junction by an external potential. In terms of performance, depletion region actuators fall between electrostatic actuators, where a physical gap separates the charges on two electrodes, and piezoelectric actuators, where the separation between the charges is on the order of lattice constants of the material. An analytic model of depletion region actuator response to an applied potential is developed and verified experimentally. The prototype micro-mechanical device utilized the local stresses produced by the depletion region actuators to generate mechanical vibrations at frequencies far below the resonance frequencies of the structure. A laser Doppler vibrometer was used to measure and compare the displacements and vibration patterns caused by the depletion region and electrostatic actuators. Utilizing depletion region actuators neither requires etching of narrow gaps, which is technically challenging nor is there a need for introducing piezoelectric materials into the fabrication process flow. The simple operating principle and the possibility of exploiting the technique for various optimized linear or nonlinear actuation at small scales provide opportunities for precise electro-mechanical transduction for micro- and nano-mechanical devices. These actuators are therefore suited for the co-fabrication of micro- and nano-mechanical systems and microelectronic circuits. Additionally, the produced strains depend only on the depletion region specifications and the excitation voltage and do not scale with device dimensions. As such, depletion region actuators can be candidates for efficient nanoscale electromechanical actuation.

## Introduction

Improvements in the performance of microsystems often necessitate the integration of the micromechanical structures and microelectronic circuits^1^. Two approaches are presently pursued by the developers of such systems: (a) Chip-level integration within a sub-optimal manufacturing technology based on compromises between micromechanical and microelectronic fabrication technologies; and (b) System-in-package integration, with separate dies for the Micro-Electromechanical Systems (MEMS) and Integrated Circuit (IC) components with compromises on size, performance, and cost. While the microelectronic manufacturing industry is essentially based on fabricating Complementary Metal Oxide Semiconductor (CMOS) transistors, the micromechanical devices mainly employ electrostatic actuation (based on Coulomb force between charged objects) and sensing (based on measurement of changes in capacitance)^2^. This is due to the simplicity of the electrostatic transducers that results in simpler manufacturing^3^. In case of integrated CMOS + MEMS chips, the actuation mechanisms are primarily limited to electrostatic and thermal transductions as the process complexity caused by techniques such as piezoelectric transduction requires compromise on the microelectronic manufacturing. On the other hand, fabrication of MEMS is sensitive to stress gradients in structural layers. This further complicates the design of MEMS devices in microelectronic processes where the processes are often optimized for the electrical responses of the layers.

Despite the widespread applications, electrostatic transduction is not efficient at converting energy between the electrical and mechanical domains, which, for instance, increases the signal losses through resonant microdevices used in timing, filtering, and resonant sensor applications^4^. The transmission losses can be lowered through the coupling of several resonators and operating them in parallel at the expense of design complexity, size, and yield^5^. The most effective method to improve electromechanical coupling efficiency for single devices, however, has been the reduction of the electrostatic gap to sub-micron levels through structural layers that can be up to tens of microns thick^6,7^. Realizing such narrow gaps not only is technically challenging, but it also compromises the linear dynamic range of these devices. It is possible to fill the gap with a material with high dielectric constant to improve the electrostatic transduction efficiency^8,9^. While helping with the transduction efficiency, the introduction of several material interfaces leads to increased process complexity and device losses while still requiring the etching of narrow gaps. On the other hand, as the structural dimensions of micromechanical devices are decreased, electrostatic transduction becomes less efficient as the minimum gap between the structures is limited by the manufacturing technology which often does not scale proportionally with other device dimensions.

Piezoelectric transduction has been employed to address some of the aforementioned challenges^10,11^. At atomic scales, the basic principle of operation is similar to electrostatic devices as the application of an electric field leads to the displacement of electrical charges in piezoelectric materials. However, the separation between the opposite charges is about the lattice constant of the crystal (i.e., about 1 nm) in contrast to the physical gap between the electrodes of electrostatic transducers that is typically on the order of 0.5–5 µm. The result is a significantly higher electromechanical coupling coefficient. Piezoelectric materials are employed where low transmission losses are needed, such as in quartz timing resonators and Film Bulk Acoustic Resonator (FBAR) filters. Several research groups have developed technologies to use thin films of piezoelectric materials for signal transduction while the main structure is made from silicon^11,12^. While the use of piezoelectric layers is gaining momentum between foundries for MEMS, chip-level integration of piezoelectric MEMS and electronics is currently not possible through MEMS + IC foundries, which typically are quite restrictive regarding the materials permitted in their processes due to cross-contamination concerns.

It has been shown that thermal actuators may be used at small scales for the conversion of electrical signals to mechanical displacements at high frequencies^13,14^. Thermal micro-actuators can be made from a single layer of silicon among other materials available in a process. However, thermal actuators chiefly utilize Joule heating, and therefore, are considered *power-hungry*, often consuming significantly more power per device compared to electrostatic or piezoelectric actuators. As device dimensions are decreased, the heat losses through device anchors to the substrate will result in further inefficiencies in electro-mechanical transduction for these devices.

There is thereby a need for developing new techniques for scalable and efficient transduction at the micro- and nano-scales. The use of *pn* junctions for actuation was first proposed in the 1960’s for piezoelectric semiconductors, but technical challenges did not allow for practical utilization of the concepts^15,16^. Proof of principle devices on silicon have been studied by different research teams where the actuators were coupled to mechanical resonators for easier signal detection^17–20^. The depletion region actuators (DRA) are suitable for integrating with conventional semiconductor processes as they do not complicate the manufacturing process. Since the actuator is made entirely of silicon, it is possible to fabricate high quality-factor resonators due to low material losses. Whenever *pn* junctions can be produced in a fabrication process, one may take advantage of various coupling mechanisms between the mechanical and electronic domains (e.g., piezo-junction or piezo-avalanche effects) for the detection of structural deformations and producing feedback signals as needed^21–23^. Lastly, the size of the actuator can be as small as the lithographical resolution or as thin as the depletion region itself in stacked layers in a standard process. On the other hand, the actuators can be constructed such that they predominantly produce lateral or normal strains within the same fabrication process. Coupled with the possibility of local actuation, this allows for schemes for optimized actuation at small scales. Earlier models for the DRA either treat the junction as a basic capacitor or have been developed for particular resonant modes with significant reliance on measured device-specific parameters to match experimental results^17,20,24,25^. In this paper, we present a general, experimentally verified model for the actuator operation that is valid for the off-resonance operation of the devices, and hence, can be applied to developing optimized actuators for micro- and nano-systems.

## Results and Discussion

### Operating principles

The operation of DRA is based on the net space charge that exists within the depletion region of a *pn* junction. At the interface between the *p* and *n* sides of a junction, the majority carriers (i.e., holes and electrons, respectively) diffuse into the opposite side of the junction, leaving behind ionized donors and acceptors that are fixed within the lattice. As a result, a region with a net space charge that is depleted of mobile charge carriers forms at the interface between the two sides of the junction (i.e., the depletion region). The space charge in the depletion regions produces an internal electric field, leading to (1) A built-in voltage-drop across the junction; and (2) An attractive body force between the two sides of the junction. The width of the depletion region, and thus the force across it, can be modulated by an external voltage, *v*_*Act*_. *PN* junctions resemble electrostatic actuators when viewed in terms of the attractive force between opposite charges. For the typical levels of doping, the distance between the fixed charges is 10’s to 100’s of nm, making DRA similar to piezoelectric transducers. In terms of linearity, DRA indeed fall between electrostatic and piezoelectric actuators as follows^19^.

**Figure 1:**
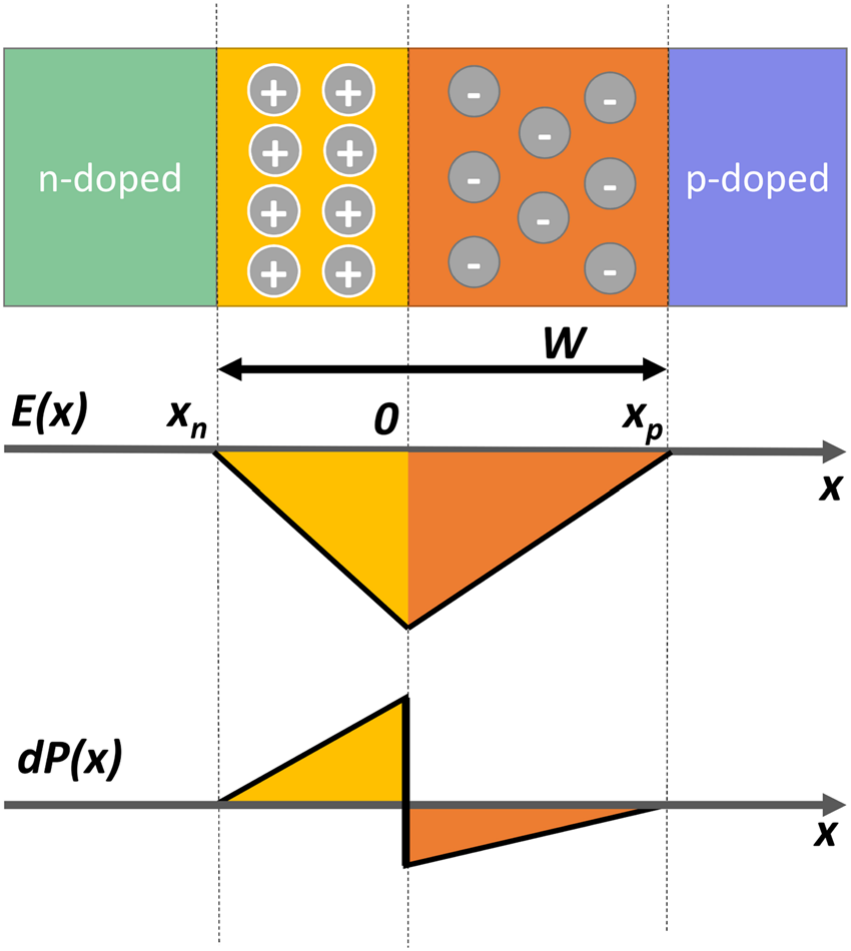
Depletion region structure and distribution of electric field (E) and internal stress (dP) inside the junction.

Applying an external voltage to the *pn* junction affects the equilibrium in carrier distributions and results in changing the depletion region width. A reverse bias will increase the depletion region width while a forward bias reduces the width and can lead to the disappearance of the depletion region when large currents flow through the junction. Setting the location of the junction (i.e., where the concentrations of acceptors, ${N}_{a}(x)$, and donors, ${N}_{d}(x)$, are equal, or ${N}_{a}(0)={N}_{d}(0)$) as the origin, the space charge distribution across the depletion region for a one-dimensional model is given by:

1 $$dQ(x)=q({N}_{d}(x)-{N}_{a}(x))A$$

where *q* is the elementary electrical charge and *A* is the junction area while *x* varies between the edges of the depletion region (i.e., $-{x}_{n} < x < +{x}_{p}$, where *x*_*n*_ and *x*_*p*_ are the width of the depletion region on the *n*- and *p*-sides of the junction, respectively). The electric field within the depletion region can be found from Gauss’s law:

2 $$E(x)={\int }_{-{x}_{n}}^{x}\frac{dQ(x)}{{\epsilon }A}dx$$

where ${\epsilon }$ is the permittivity of the semiconductor (see Fig. 1). The built-in potential, *V*_*BI*_, across the depletion region can be found from integrating the electric field, which will be the same as the difference between the Fermi levels at the edges of the depletion region^26^:

3 $${V}_{BI}=-\,{\int }_{-{x}_{n}}^{{x}_{p}}E(x)dx=\frac{{k}_{B}T}{q}\,\mathrm{ln}(\frac{{n}_{{x}_{n}}}{{n}_{{x}_{p}}})$$

where *k*_*B*_ is the Boltzmann’s constant, *T* is the temperature, and ${n}_{{x}_{p}}$ and ${n}_{{x}_{n}}$ are the electron concentrations at the *p*- and *n*-side edges of the depletion region, respectively. For a varying doping profile, the electron concentrations can be found from ${n}_{{x}_{n}}={N}_{d}(-{x}_{n})-{N}_{a}(-{x}_{n})$ and ${n}_{{x}_{p}}={n}_{i}^{2}/({N}_{a}({x}_{p})-{N}_{d}({x}_{p}))$, where *n*_*i*_ is the intrinsic carrier concentration of the semiconductor. The stress on a slice of the junction is found from:

4 $$dP(x)=\frac{dQ(x)}{A}E(x)$$

Equation (4) can be used to calculate the displacement distribution by calculating the compression of the material where this stress is axially applied to the block of material with a width of $x$:

5 $$u(x)={\int }_{-{x}_{n}}^{x}\frac{dP(x)}{Y}x\,dx$$

where *Y* is the elastic modulus of the semiconductor which is assumed to be independent of the variations in doping levels. Finally, the strain across the depletion region may be found from

6 $$\varepsilon (x)=\frac{du(x)}{dx}$$

**Figure 2:**
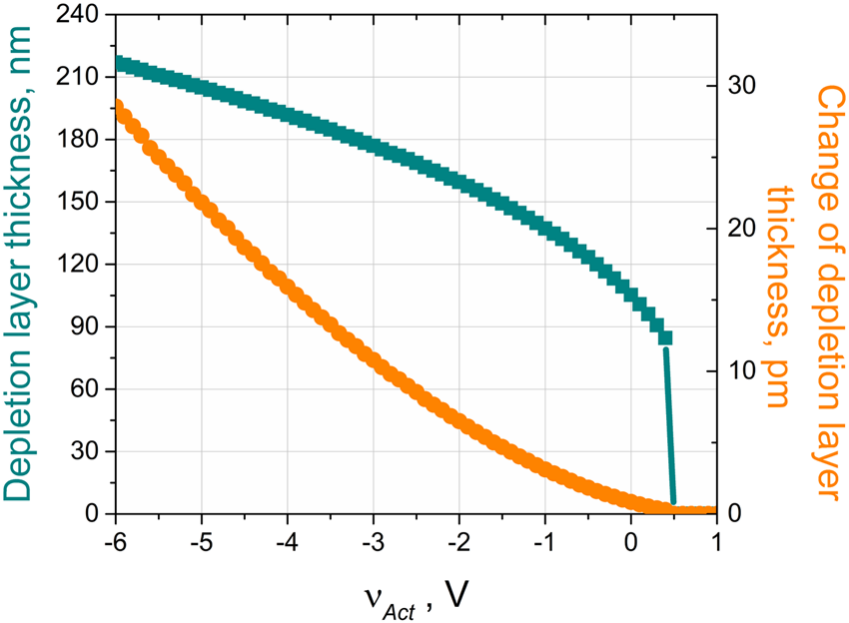
Variations in the depletion region width with an applied bias voltage. Note that the depletion region, and hence the strain across it, vanish with the heavy injection of carriers under the forward-bias conditions.

These equations along with the assumption of charge neutrality across the depletion region (i.e., ${\int }_{-{x}_{n}}^{{x}_{p}}dQ(x)dx=0$) can be solved for arbitrary doping profiles to calculate the induced strains. The depletion region width, and thus the strains, can be modulated with an externally applied voltage. Particularly in the reverse bias region, the field produced by an external voltage adds directly to the internal electric field of the junction and the depletion region boundaries move deeper inside the *p*- and *n*- sides (i.e., the depletion region widens with reverse voltage). As the position of junction boundaries depend on the local doping concentrations, the changes in the locations of depletion region edges under the external electric potential could be different. Figure 2 illustrates the dependence of the depletion region width on the external voltage and the resulting displacement of the boundaries. This result is obtained by numerically solving Eqs 1–6 using the doping profiles for the devices that are later discussed in this paper (see the *Materials and Methods* section for details).

Simple expressions can be derived for an abrupt junction with uniform dopant concentrations (i.e., ${N}_{a}(x)={N}_{a}\,H(x)$ and ${N}_{d}(x)={N}_{d}\,H(-x)$ where *H*(*x*) is the Heaviside step function) that is reverse biased with an external voltage of *v*_*act*_. In this case, the depletion region width is given by^26^:

7 $${W}_{0}={x}_{n}+{x}_{p}=\sqrt{\frac{2{\epsilon }}{q}(\frac{1}{{N}_{a}}+\frac{1}{{N}_{d}})({V}_{BI}+{v}_{act})}$$

The internal strain within the depletion reduces this initial width by *u*(*x*_*p*_):

8 $$u({x}_{p})=-\,\frac{\sqrt{2{\epsilon }q}}{3Y}\sqrt{\frac{{N}_{a}{N}_{d}}{{N}_{a}+{N}_{d}}}{({V}_{BI}+{v}_{act})}^{\frac{3}{2}}$$

This result further establishes the DRA between the piezoelectric and electrostatic actuators, where the relationships between the displacements and applied potentials are linear and quadratic, respectively.

**Figure 3:**
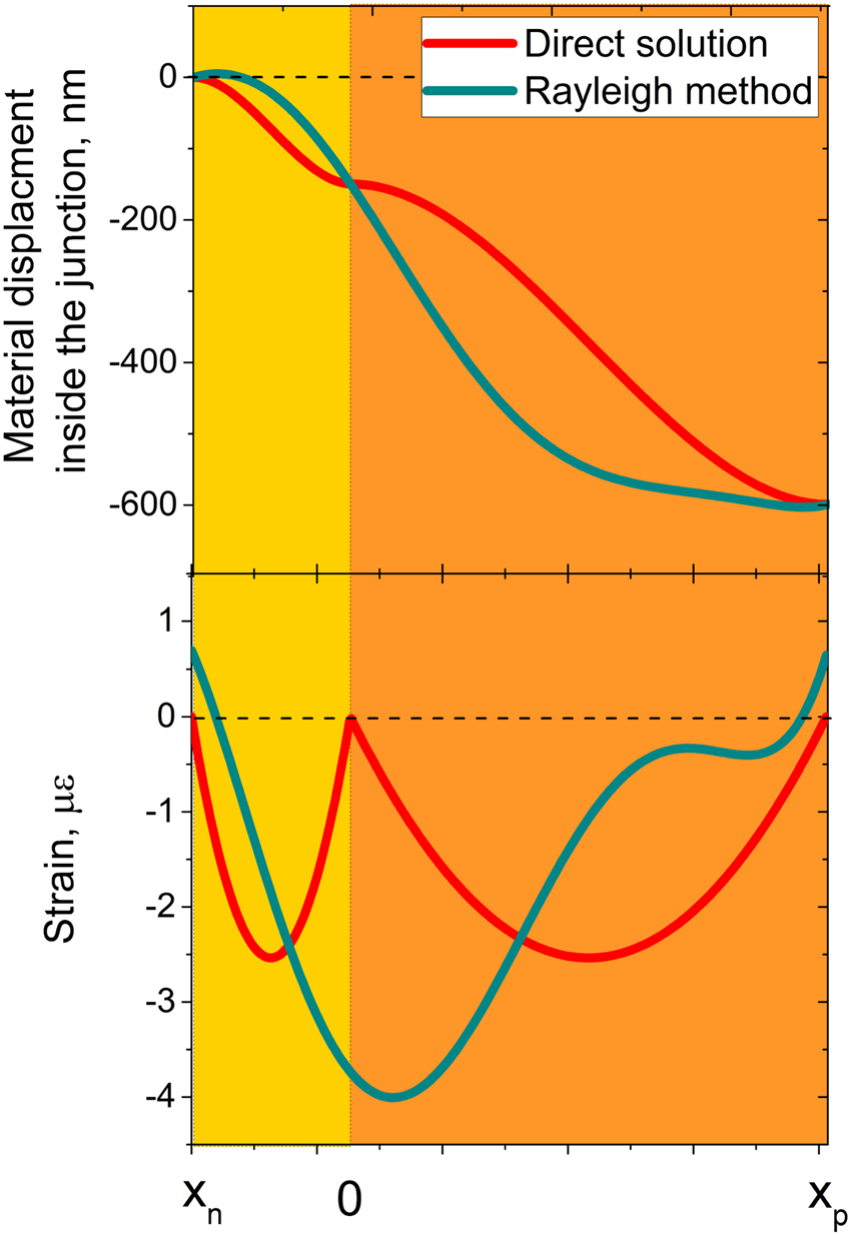
Comparison of the local displacement and stain inside the depletion region calculated through the exact and approximate methods.

Energy-based methods, such as the Rayleigh-Ritz method, are often used to study the response of various mechanical structures. These techniques typically assume the solution to be of a polynomial trial function and minimize the energy in the system to calculate the coefficients. Popular numerical analysis methods such as Finite Element Analysis (FEA) are in fact based on energy methods. These methods can be used to estimate the total displacement within the depletion region under the internal electrostatic forces^27^. However, while the estimated total displacements may be close to the actual solutions, limited by the assumptions of trial functions, these approaches do not provide a correct understanding of the mechanisms and features. Figure 3 compares the local displacements for an abrupt junction calculated based on the Rayleigh-Ritz method against the exact solution. While the total estimated displacement is close to the exact solution, it can be seen that the displacement field and strains are considerably different.

### Experimental results

The strains across a depletion region can be modulated to create mechanical vibrations of a coupled structure. The demonstrated examples thus far couple *pn* actuators to microresonators with high quality-factors to excite various vibration modes^18,20,25,28,29^. Employing microresonators allows for the natural amplification of the structure response by the quality-factor of the structure, which can range from 100’s to 10,000’s, producing vibrations that can be detected more easily. The drawback is that quality factor, especially at small scales, is affected by various factors and cannot be measured accurately. The developed analytic solutions to study the response of these structures often use the measured quality-factors to compare the results from models and experiments. In this work, we designed a micromechanical structure that allowed us to investigate the device performance off-resonance, and hence, could be used to verify the presented model.

**Figure 4:**
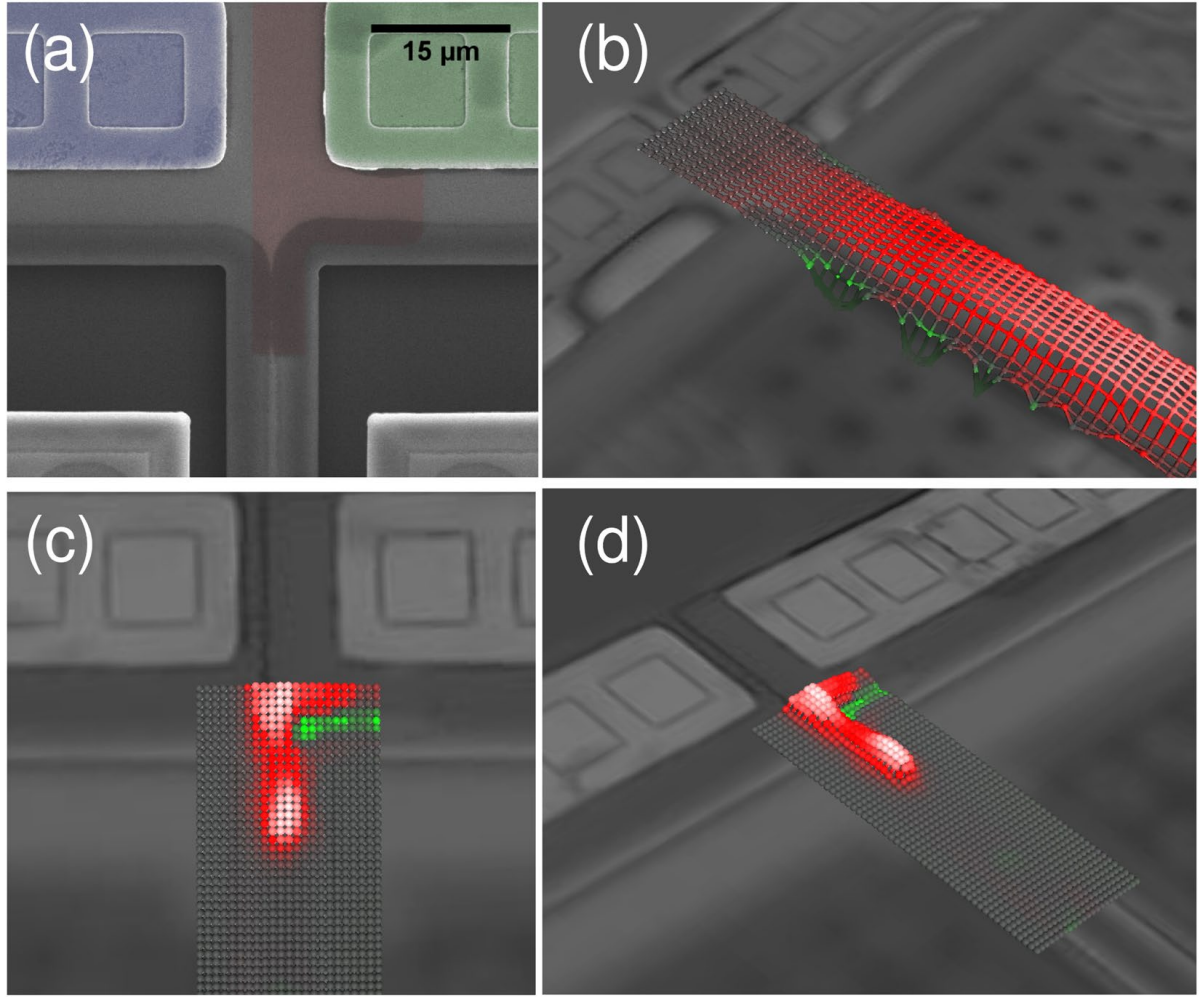
(**a**) The SEM image of the DRA at the base of the released beam; (**b**) Beam vibrations due to electrostatic actuation by applying a voltage to the substrate; and Top (**c**) and side (**d**) views of the displacements caused by applying the excitation voltage to the DRA. See the Supplementary video clips for the animated vibration pattern measurements.

To investigate the behaviour of a *pn* junction under an applied electric potential, a DRA was embedded at the anchor point of a clamped-clamped beam (300 *μm* long and 10 *μm* wide). The *pn* junction is placed where the clamped-clamped beam is anchored to the substrate in order to generate larger displacements for produced strains. Two rectangular winglets (270 *μm* × 140 *μm*) were connected to the center of the beam to simplify the optical and capacitive measurements of the displacements. Figure 4 illustrates the close-up views of the DRA and the beam.

Figure 4a shows a top view of the base of the fabricated device where portions of the main beam, the embedded *pn* actuator, and the metal contacts to the *p* and *n*-doped areas are shown. The *pn* junction (highlighted in red) is around 6 *μm* wide and is connected to the metal pads (highlighted in green for the *n*-side and purple for the *p*-side) away from the device edge. The fabricated structure can be electrostatically forced into vibrations by applying a voltage to the substrate. Structural displacements were measured using a laser Doppler vibrometer (LDV) with a movable device stage. A measurement grid was produced near the anchor of the device that was slightly wider than the fabricated beam. The LDV measured the spot displacements across the grid by moving the stage in 0.8 *μm* steps and synchronizing them by locking them to the excitation signal. With electrostatic actuation from the substrate, the entire beam experiences a distributed electrostatic force and responds accordingly. Figure 4b shows the vibration pattern of the beam superimposed on the stationary image of the device. On the other hand, Fig. 4c,d show the top and side views of the local deformations of the doped region when the excitation voltage was applied across the junction (the beam and substrate where both held at ground potential). Animated data of LDV measurements for the two excitations are provided as supplementary data for reference.

**Figure 5:**
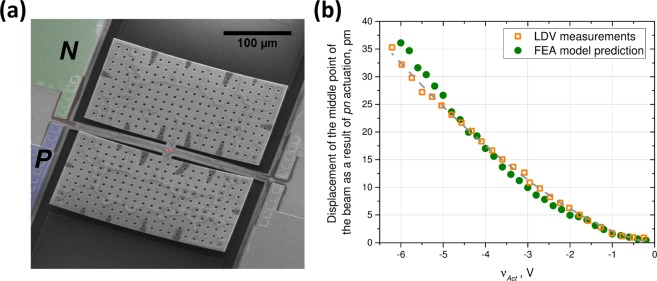
(**a**) SEM image of the entire device; and (**b**) Comparison of measured and predicted displacements at the centre of the beam with DRA. The fitted line to LDV measurements is proportional to ${{v}}_{{act}}^{1.514}$ which is in good agreement with the theoretical prediction of ${{v}}_{{act}}^{3/2}$.

To demonstrate the utility of the local deformations of the *pn* junction for mechanical actuation, an excitation signal in the form of ${v}_{act}(1+\,\sin (2\pi {f}_{exc}t))$ was applied to the junction so that the junction was reverse-biased at all times. The displacements at the centre of the beam were calculated in a finite element solver by applying the corresponding strains found from the numerical solution of Eqs 1–6 using the doping profile for the fabricated devices. The device response was then measured experimentally. During the experiments, the value of *v*_*act*_ was changed from −3V to 0 V with *f*_*exc*_ = 5 *kHz*, well below the device fundamental resonant frequency of ~30 kHz. The displacements were measured at a single spot in the middle of the beam (see Fig. 5a) as it exhibits the largest vibration amplitude, and hence, the largest signal-to-noise ratio. Figure 5b illustrates the good match between the measured signal amplitudes against the predicted results from our model, with the difference between the experimental and calculated values being less than 10% in all cases. As can be seen, increasing the amplitude of the excitation signal leads to a nonlinear increase in the vibration amplitudes. The best polynomial fit for the relationship between the displacements and input voltage amplitude resulted in a power of 1.514 (i.e., $\Delta y\propto {v}_{act}^{1.514}$), which is in excellent agreement with Eq. (8) that predicts a response proportional to ${v}_{act}^{3/2}$.

## Conclusions

In this report, we demonstrated the utility of DRA for localized deformations on micromechanical structures. These actuators rely on the natural formation of a depletion region at a *pn* junction and the resultant internal electric field that leads to the development of internal stresses and strains. Coupled to a proper mechanical structure, modulation of these internal strains with external voltage signals can be utilized to produce sizable displacements at the micro- and nano-scales. A simple, physics-based model was developed for these actuators whose validity was confirmed experimentally. This model was employed to calculate internal strains at the base of a beam, which in turn were used to estimate the deflections at the centre of the beam. Excellent agreement was found between the predicted and measured results. Noting that the developed model is based only on the physical properties of the material and not the mechanical structure, it can be used to micro- and nano-systems with embedded DRA for optimal response. By eliminating the dependence on narrow gaps, reducing the sensitivity to internal stresses and stress-gradients in structural layers, and developing actuators based on standard materials, DRA-based systems can simplify the MEMS + IC integration. On the other hand, the DRA response is unaffected as the device dimensions are scaled down as long as the depletion region remains embedded within the structure. This further provides opportunities to utilize the DRA for efficient electromechanical coupling at the nano-scale.

## Materials and Methods

### Device fabrication process

**Figure 6:**
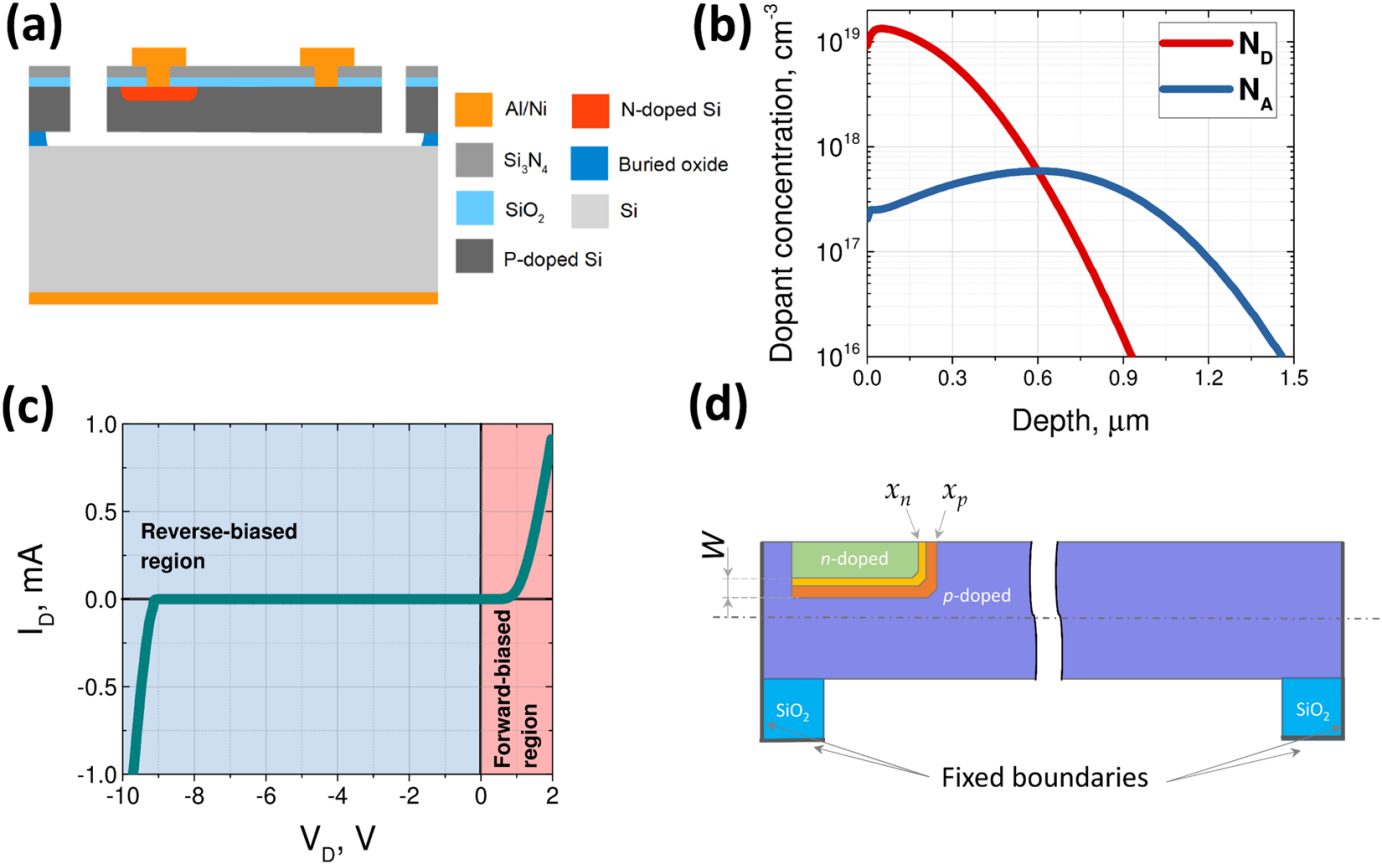
(**a**) Cross-section of the wafer through a device; (**b**) Doping profile for the junction; (**c**) Measured Current-Voltage characteristic curve for the fabricated junction; and (**d**) Cross-section of the device structure used for FEA simulations.

The cross-section of the fabricated devices is shown in Fig. 6a. The design prototype was fabricated through a bulk micromachining process from the 2 *μm* thick device layer of a silicon-on-insulator (SOI) wafer above a 2 *μm* buried oxide (BOX) layer. Boron-ions were implanted on *p*-doped silicon device layer to bring up the background doping concentration from $\sim {10}^{15}\,{\rm{to}}\,\sim {10}^{17}\,{\rm{atoms}}/{{\rm{cm}}}^{3}$, lowering electrical resistivity to the range of 0.1–0.2 Ω. *cm*. An oxidation process concurrently was employed to drive boron ions to a depth of ~1.2 *μm* and grow 230 nm of thermal oxide as the mask layer for the subsequent doping process. Phosphorus-ions were implanted on the boron-doped silicon through openings in the aforementioned oxide layer such that *pn* junctions were formed at a depth of ~600 nm after annealing. The final doping profiles for boron and phosphorous, which are also used for the modelling of the device behavior, are shown in in Fig. 6b. The wafer surface was then coated with a 50 nm thermal oxide followed by a 200 nm thick, low-stress silicon nitride (Si_3_N_4_) film, through low pressure chemical vapor deposition (LPCVD). This layer is needed as the passivation layer for electrical isolation and minimization of junction leakage current. The nitride layer was then patterned to provide access to the underlying silicon through vias. A 200 nm layer of aluminum-silicon alloy (Al_0.99_Si_0.01_) topped with 100 nm nickel (Ni) was then deposited through lift-off process for electrical connection to both sides of the junctions. The nickel layer protected the aluminum layer during the final sacrificial release step. A similar metal layer stack was deposited on the back side of the wafer after the removal of the of the dielectrics to allow electrical contact to the substrate. The device layer was then patterned through a deep reactive ion etch (DRIE) process after removal of the nitride layer through a reactive ion etching (RIE) step. Finally, the buried oxide was removed using vapor hydrofluoric acid (VHF) through the gaps and etch holes distributed across wider structures to release the micro-structures. The fabricated devices were wire-bonded within 44-pin ceramic packages for testing and characterization.

### Device characterization

The current-voltage characteristics of the fabricated *pn* junction measured using a Keithley 2400 source-measure unit is shown in Fig. 6c.

Scanning electron microscope (SEM) images were taken using a field emission scanning electron microscope (FEI Nova NanoSEM 430) at 4D LABS, SFU. Chips were attached to the sample holders and investigated without any special pretreatment.

The mechanical response of the packaged devices was then characterized using a Polytec MSA-050 Laser Doppler Vibrometer (LDV). The apparatus allows performing displacement measurements with frequencies in the range from DC up to 20 MHz using a Helium-Neon laser with a wavelength of 633 nm. The scanning resolution was limited by the optical system and was just under 1 *μm* while the motorized stage allows performing mapping with a resolution of 250 nm. To remove the influence of the environmental vibrations and decrease the noise level, the LDV was placed on a vibration isolation platform. The noise level in the investigated region was around 300 fm. During the tests, the package was fixed to the motorized stage. The devices were connected to the DC and AC voltage sources through direct connections to the package. Measurements with the LDV could be performed at a spot (Fig. 5b) or on a grid (Fig. 4b–d).

For electrostatic actuation experiments, the excitation voltage (1.5 V DC + 1.5 AC in form of either a sine wave or a chirp) was applied to the substrate through the connections to the backside of the wafer. The *p*- and *n*-sides of the junction on the beam were short-circuited together and connected to ground.

For *pn*-junction actuation, the substrate and *p*-side of the junctions were connected to ground to minimize the potential for electrostatic actuation. To voltage on the *n*-side was a combination of an AC component for dynamic excitation and a DC component to ensure the junction remained reverse-biased throughout the experiments. A sinusoidal signal was used as the AC signal to evaluate the frequency response of the structure when needed.

### Simulation of device behaviour

The schematic illustration of a cross-section of the structure are shown in Fig. 6d. Once the internal stresses were calculated, they were used in a finite element model using COMSOL Multiphysics. For the finite element simulations, the structural material properties were those of silicon in <110> direction, including an elastic modulus of 169 GPa, Poisson’s ratio of 0.226, and density of 2330 kg/m^3^ ^30^. The 3D structure of the microdevice was replicated in the software and suitable boundary conditions were applied (thick lines in Fig. 6d). For each value of a voltage applied to the DRA, the positions of the junction boundaries *x*_*p*_ and *x*_*n*_ as well as the generated internal forces between them was calculated using the model described in the paper and applied directly to the structure. Due to the 3D structure of the actual structure, there are two perpendicular DRAs at the bottom as well as sidewalls of the junctions. However, for the large and, narrow junction we had in our structure, the displacements at the centre of the beam can mostly be attributed to the compression of the material normal to the plane of the wafer and actuating the elastic beam through the corresponding deformations (related to each other by Poisson’s ratio).

## Supplementary information

Supplementary Information

Supplementary Information

Supplementary Information
