# Supplementary material for: Localized Mechanical Actuation using pn Junctions
Source: Sci Rep. 2019 Oct 16;9:14885. doi: 10.1038/s41598-019-49988-z (PMC6795996; doi:10.1038/s41598-019-49988-z)

Localized Mechanical Actuation using *pn* Junctions: Supplementary information on fabrication process

Mikhail Kanygin, Abbin Perunnilathil Joy, and Behraad Bahreyni *

School of Mechatronic Systems Engineering, Simon Fraser University, Surrey, BC, V3T 0A3, Canada

*Author to whom correspondence should be addressed: behraad@ieee.org

The device prototypes were fabricated in a bulk micromachining process at SFU 4D LABS and Engineering Science Cleanroom Facility. A 4" Silicon-On-Insulator (SOI) wafer was used as the starting substrate with silicon crystallographic orientation along [100] direction. The device layer was chosen to be 2 μm in thickness and 1-20 $\Omega.cm$ electrically resistive. A 2 μm thick buried oxide layer was used as the sacrificial layer for final releasing of the structure. The major steps in fabrication process flow contains: 1) blanket doping (boron), 2) selective doping (phosphorus), 3) vias and metal interconnections, 4) device layer patterning, and 5) sacrificial releasing.

Process description	Schematic
**Starting substrate**<br><br>4" double side polish SOI wafer<br>Device layer: (100) Silicon, 2 μm, 1-20 $\Omega.cm$<br>Buried oxide layer: 2 μm<br>Handle layer: (100) Silicon, 500μm, 1-20 $\Omega.cm$	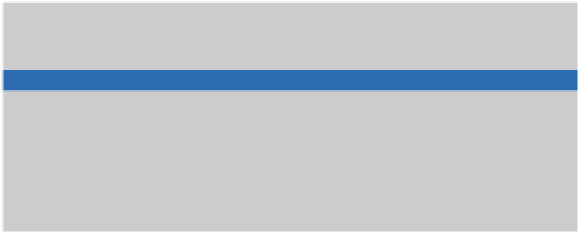
**Blanket doping – Boron**<br><br>Boron was implemented onto the entire top surface of the wafer followed by annealing to lower the electrical resistivity of device layer to the range of ~0.1 $\Omega.cm$.<br>The energy and dose of process were controlled to diffuse dopants to a depth of 1.2 μm.	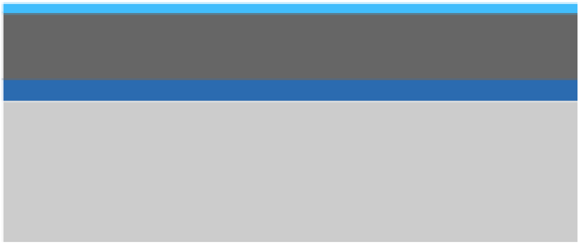
**Selective doping – Phosphorus**<br><br>A silicon dioxide layer is thermally grown and patterned for use as a masking layer for the subsequent phosphorus ion implantation and annealing. The oxide layer was then removed from the surface.<br>The *pn* junction was formed at a depth of ~600 nm from surface.	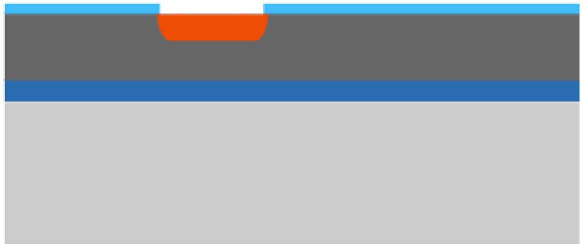

**Vias and metallization**<br><br>Thermally grown silicon dioxide and low stress silicon nitride were used as passivation layers for electrical isolation between chip areas. These layers were patterned using Reactive Ion Etching (RIE) open vias to the underlying silicon.<br>A bilayer of $Al_{0.99}Si_{0.01}$ (200 nm) and Nickel (100 nm) were thermally evaporated for contacts and surface protection for the following release step.	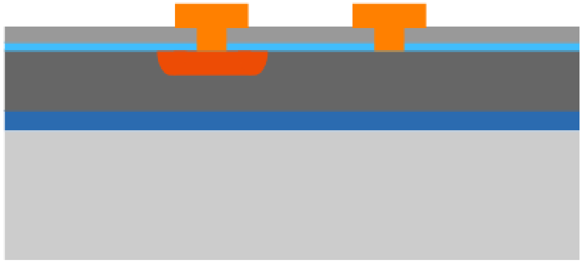
**Device layer patterning**<br><br>The nitride and silicon dioxide layers were etched by RIE to expose the silicon layer. The device layer is patterned to buried oxide layer by deep reactive ion etching (DRIE) for the definition of the mechanical structures.<br>The backside of the chip is coated with a thin aluminum layer after the removal of the dielectrics to provide electrical access to the handle layer.	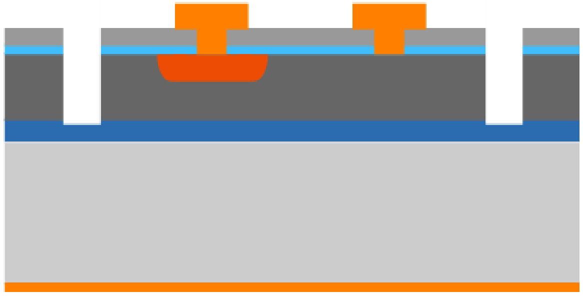
**Sacrificial release**<br><br>The oxide layer buried underneath the device layer is removed using vapor HF through openings created during the DRIE step.	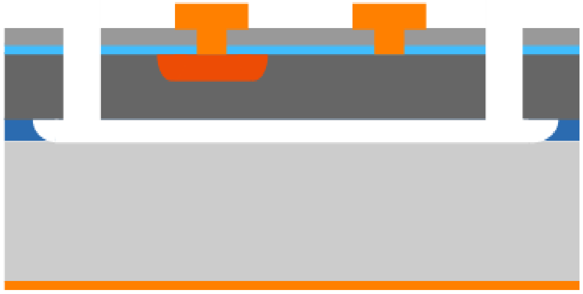

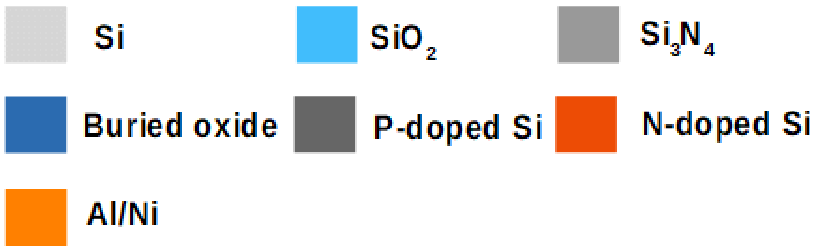

Supplement: Supplementary file 1 — Supplementary Information [file 41598_2019_49988_MOESM1_ESM.pdf]
